# Supplementary material for: Grip Strength as an Indicator of Health in Elderly Females
Source: Healthcare (Basel). 2025 May 12;13(10):1127. doi: 10.3390/healthcare13101127 (PMC12111805; doi:10.3390/healthcare13101127)
Supplement: Supplementary file 1 [file healthcare-13-01127-s001.zip › Supplementary table.pdf]

Supplementary data

Supplementary Table S1. Association of exercise and obesity

		B	SE	Wald	*p*	OR	95% CI	
							LLCI	ULCI
Step 1	Exercise or non-exercise	-0.524	0.237	4.879	*0.027	0.592	0.372	0.943
	Constant	1.567	0.204	58.931	0.000	4.793		
Variable(s) entered on step 1: Exercise or non-exercise								

B: beta coefficient

SE: standard error

OR: odds ratio

LLCL: Lower limit of the confidence interval

ULCI: Upper limit of the confidence interval

Supplementary Table S2. Association of hand grip strength and obesity

		B	SE	Wald	*p*	OR	95% CI	
							LLCI	ULCI
Step 1	Grip strength	-0.056	0.023	5.938	*0.015	0.946	0.904	0.989
	Constant	2.456	0.535	21.105	0.000	11.657		
Variable(s) entered on step 1: Grip strength								

Supplementary Table S3. Association of gait and obesity

		B	SE	Wald	*p*	OR	95% CI	
							LLCI	ULCI
Step 1	Gait	0.178	0.085	4.455	0.035	1.195	1.013	1.411
	Constant	-0.018	0.573	0.001	0.975	0.982		
Variable(s) entered on step 1: Gait								
